# Clinicopathological review of 156 appendicectomies for acute appendicitis in children in Ile-Ife, Nigeria: a retrospective analysis

**DOI:** 10.1186/s12873-015-0030-9

**Published:** 2015-05-09

**Authors:** Talabi O Ademola, Sowande A Oludayo, Olowookere A Samuel, Etonyeaku C Amarachukwu, Komolafe O Akinwunmi, Adejuyigbe Olusanya

**Affiliations:** Department of Surgery, Obafemi Awolowo University/Obafemi Awolowo University Teaching Hospital, Ile-Ife, Nigeria; Department of Community Health, Obafemi Awolowo University, Ile-Ife, Nigeria; Department of Morbid Anatomy and Forensic Medicine, Obafemi Awolowo University, Ile-Ife, Nigeria

**Keywords:** Acute, Appendicitis, Children, Late presentation, Outcome

## Abstract

**Background:**

Acute appendicitis is one of the most common causes of acute abdomen in children. Late surgical intervention is often associated with increase morbidity and sometimes fatal outcome. We sought to determine the pattern of presentation of acute appendicitis, and the effect of late presentation on surgical outcome in children.

**Methods:**

This is a retrospective descriptive study done at the paediatric surgical unit of Obafemi Awolowo University Teaching Hospitals Complex, Ile-Ife, Nigeria. The hospital records of all 180 patients (15 years and below) treated for acute appendicitis, between January 1995 and December 2012, were reviewed; only 156 patients had adequate records out of which 139 cases confirmed histologically as having appendicitis were analyzed.

**Results:**

There were 80 (57.6%) females and 59 (42.4%) males. The age range was 5-15years with mean (SD) age of 11.2 (±2.9) years. Most patients (64.7%) were more than 10 years old. Sixty-four (46%) patients had simple appendicitis while 75 (54%) patients had complicated appendicitis. More children with complicated appendicitis (63, 84.0%) presented after 24 hours of abdominal pain; and they had more vomiting (59, 78.7%), spent longer days on admission (57, 76.0%) and had more post- operative complications (34, 45.3%) compared with uncomplicated appendicitis (25, 39.1%; 29, 45.3%; 7, 10.9%; 1, 1.6% respectively), and this was statistically significant (p < 0.05). No mortality was recorded among these children.

**Conclusion:**

Late presentation was common and was associated with longer duration of hospital stay and high morbidity. No mortality was recorded from the disease.

## Background

Acute appendicitis is the most common cause of abdominal pain requiring emergency surgical intervention in children and adolescents [1]. It is generally uncommon in toddlers and rare during infancy [1-3]. This condition once thought to be rare in Nigeria, is now believed to be on the increase in many urban centers due to changes in life style and diet [4,5]. It is caused either by obstruction of the appendiceal lumen by a faecolith or lymphoid hyperplasia from primary (bacterial or viral) infection of the appendix [6,7]. In our hospital, as in most parts of sub-Saharan Africa, the diagnosis of acute appendicitis in children is mainly clinical; supported by laboratory tests like full blood count (complete blood count) with emphasis on the white blood cell count and its differential count). Ultrasonography (for the inflamed appendix) is sometimes done to aid diagnosis especially in females. Computerized tomography scan of the abdomen and laparoscopy are rarely used for diagnostic purposes as the facilities are not readily available or expensive. Early presentation and diagnosis, coupled with prompt surgical intervention are major determinants of best treatment outcomes [5,8,9]. Diagnosis on clinical grounds is often challenging as many disorders of the digestive tract may mimic acute appendicitis especially in children. Thus indiscriminate resort to early surgery in all patients diagnosed of acute appendicitis on purely clinical grounds could lead to a relatively high negative appendectomy rates. This has necessitated the use of white blood count (and its differential count) and C – reactive protein value in improving diagnostic yield. The high morbidity and occasional mortality associated with acute appendicitis are related to delay in presentation by patients or delay in diagnosis by the clinician. These delays may result in complications like gangrene, perforation, appendiceal mass and peritonitis [2,3,5], all of which would prolong hospital stay and increase the cost of treatment [5]. The aim of this study is to determine the pattern of presentation of children with acute appendicitis, to correlate the effect of late presentation on surgical outcome and to determine rate of negative appendicectomy amongst children in Ile-Ife, Nigeria.

## Methods

This is a retrospective review of children aged 15 years and below who had appendicectomy for suspected acute appendicitis at the paediatric surgical unit of the Obafemi Awolowo University Teaching Hospitals Complex, Ile-Ife, between January 1995 and December 2012. The study site is a tertiary referral institution but in addition offers primary and secondary health care services to people who are predominantly farmers, artisans, traders and civil servants in southwest, Nigeria.

The study was granted ethical approval by Research and Ethics Committee of the Ife Central Local Government (ERC/2008/A/03). The names and hospital number of patients within the age range 15 years and below who had appendicectomy were extracted from the theatre register. The list generated was used to retrieve the case notes of the patients from the medical records department. From these case notes, data on the demographic characteristics, location and duration of abdominal pain, frequency of vomiting, presence of anorexia, and abdominal distention were extracted. Other information retrieved included severity of fever, presence or absence of dehydration, and diarrhea. Also a review of laboratory and radiological investigations done, intraoperative findings, duration to the commencement of oral intake post-surgery, post-operative complications, duration of admission, management outcome and histopathology report of the surgical specimen were extracted and entered into a proforma. The patients’ pre-operative evaluation was largely clinical, supported by complete blood count (especially packed cell volume and white blood cell count with differential count). Prior to surgery, all patients were resuscitated with intravenous fluid, maintained on nil per os and commenced on broad spectrum antibiotics to cover for gram positive and negative bacteria as well anaerobic organisms. The antimicrobial combinations used were either metronidazole with ceftriaxone or ciprofloxacin; or metronidazole with gentamycin and ampiclox. The operations for acute appendicitis were conducted either by consultant surgeons or by senior surgical residents (senior registrars). Patients with histological report of reactive follicular hyperplasia with otherwise normal features were regarded to have had negative appendicectomy. Patients with histological features of appendicitis were divided into 2 groups: uncomplicated (simple) and complicated appendicitis based on intra-operative findings. Children with inflamed, turgid, and hyperemic appendix intra-operatively and histological features of appendicitis (inflamed appendix with neutrophil infiltration of muscularis propia) were regarded as uncomplicated appendicitis; while those who had gangrenous or ruptured appendix with or without intra-peritoneal abscess at surgery for whom the histological report of the removed appendix showed ulcero-phlegmonous, thick wall inflamed or suppurate appendix were taken as complicated appendicitis. Patients who presented after 24 hours of abdominal pain were presumed to have presented late. Children who had negative appendicectomy were excluded from further analysis when comparing simple appendicectomy with complicated ones. Data was analyzed using Microsoft excel and Statistical Package for Social Science (SPSS) version 17. The data were summarized using mean and standard deviation (SD) for continuous variables and frequencies for categorical variables. Inferential statistics with Chi-square test was used to establish association with p value less than 0.05 considered as statistically significant.

## Results

One hundred and eighty children aged 15 years and below had appendicectomy within the study period. Only 156 (86.7%) of these had adequate data for analysis. Of these, 17 (10.9%) were negative appendicectomies which were more common in females (70.1%). Data on children who had negative appendicectomy were excluded from further analysis. There were 139 (89.1%) confirmed cases of acute appendicitis in children during the study period, 80 (57.6%) girls and 59 (42.4%) boys giving a female to male ratio of 1.4 to 1.0. Their ages ranged from 5 to 15 years with a mean age of 11.2 (±2.9) years. Forty-nine (35.3%) patients were aged 5-10years while 90 (64.7%) were aged 11-15years. Appendicitis was thus more common in the second decade of life. All (100%) patients presented with history of abdominal pain. One hundred and two patients (73.4%) had migratory right iliac fossa pain while the remaining 37 (26.6%) presented with generalized abdominal pain *ab initio*. The clinical features are as highlighted in Table 1. Forty patients (28.8%) had normal temperature, 54 (38.8%) had low grade fever while 45 (32.4%) had high grade fever on admission. There were 64 (46.0%) cases of uncomplicated (simple) appendicitis; while 75 (54.0%) patients had complicated appendicitis which comprised 71 ruptured and 4 gangrenous appendices giving a perforation rate of 51.1% (71/139).

**Table 1:** **Clinical features of patients with acute appendicitis (N = 139)**

**Clinical features**	**No. of patients (%)**
**Abdominal pain**	139 (100.0)
Migratory right iliac fossa pain	102 (73.4%)
Generalized abdominal pain	37 (26.6%)
**Anorexia**	127 (91.4)
**Vomiting**	114 (82.0)
**Fever**	99 (71.2)
Normal temperature (36.6 – 37.2°C)	40 (28.8%)
Low grade fever (37.2 – 37.9)	54 (38.8%)
High grade fever (>38.0°C)	45 (32.4)
**Diarrhoea**	40 (28.8)
**Constipation**	27 (19.4)
**Abdominal distension**	25 (18.0)
**Dehydration**	35 (25.2)

Eighty-eight (63.3%) patients presented after 24 hours of onset of abdominal pain: sixty -three (84.0%) among those with complicated appendicitis versus 25 (39.1%) among those with uncomplicated (simple) appendicitis, (Table 2). There was a statistical significant relationship between duration of disease at presentation and the likelihood of the disease being complicated (X^2^ = 30.0, p = 0.001). Fifty-nine (78.7%) patients among those with complicated appendicitis had more than one episode of vomiting prior to presentation when compared with 29 (45.3%) children among those with uncomplicated appendicitis. p = 0.001.

**Table 2:** **Duration of abdominal pain, frequency of vomiting, before presentation, hydration status at presentation and operative procedure, (N = 139)**

**Variable value**		**Type of appendicitis**		**Total N (%)**	**p-value**
		**Simple (uncomplicated) N = 64 (%)**	**Complicated N = 75 (%)**		
**Duration of pain (days)**	≤1	39 (60.9)	12 (16.0)	51 (36.7)	0.001
	>1	25 (39.1)	63 (84.0)	88 (63.3)	
**Dehydration**	No	60 (93.7)	46 (61.3)	106 (76.3)	0.001
	Yes	4 (6.3)	29 (38.7)	33 (23.7)	
**Number of vomiting episodes**	None at all or once	35 (54.7)	16 (21.3)	51 (36.7)	*0.001*
	More than once	29 (45.3)	59 (78.7)	88 (63.3)	
**Operative procedure done**	Appendectomy only	61 (95.3)	3 (4.0)	64 (46.0)	0.001
	Appendectomy + drainage of localized abscess	0 (0)	25 (33.3)	25 (19.0)	
	Exploratory Laparotomy + appendectomy + drainage of intra-peritoneal abscess	0 (0.0)	45 (60.0)	45 (32.4)	
	Exploratory Laparotomy + right hemi-colectomy + drainage of intra-peritoneal abscess	0 (0.0)	2 (2.7)	2 (1.4)	
	Laparoscopic appendectomy	3 (4.7)	0 (0.0)	3 (2.2)	

Serum electrolytes and creatinine were done in 116 (83.5%) patients, out of which 68 (58.6%) had complicated appendicitis while 48 (41.4%) had simple appendicitis. Of these 116 patients, twenty patients had electrolyte imbalance. A higher proportion of patients with complicated appendicitis compared with simple appendicitis had electrolyte imbalance (18, 26.5% versus 2, 4.2%). And this showed a statistically significant difference, p = 0.002. Eighty percent of the electrolyte abnormalities were due to azotemia while the remaining 20% was due to hypokaelemia present in those with complicated appendicitis. Of the 33 (23.7%) patients who were dehydrated at presentation, 4 (6.3%) were among those with simple uncomplicated appendicitis compared with 29 (38.7%) who had complicated appendicitis, p = 0.001.

The surgical approaches used included: Lanz (84; 60.4%), transverse infra-umbilical (34; 24.4%), midline (9; 6.5%), transverse supra-umbilical (6; 4.3%), grid iron (3; 2.2%) and laparoscopic port site incisions (3; 2.2%). At surgery, the appendix was retro-caecal in position in 100 (72.0%) patients, pelvic in 18 (12.9%) patients; while pre–ileal, para - caecal, and post-ileal appendices were observed in 10 (7.2%), 6 (4.3%) and 5 (3.6%) patients respectively. The length of the appendix varied from 4 to 25 cm with a mean (±SD) of 9.0 (±2.9) cm. The operative procedures done are as shown in Table 2. Oral feeding was commenced between first and eighth post-operative day (mean ± SD = 2.4 ± 1.2 days). Most patients with uncomplicated appendicitis (59; 92.2%) were fed within 48 hours of surgery as compared to those with complicated appendicitis (34; 45.3%) and this was found to be statistically significant (χ^2^ = 34.2, p = 0.001). Overall post-operative complication and wound infection rates were more common among children with complicated appendicitis compared to simple appendicitis: (45.3% versus 1.6%, 38.7% versus 1.6% respectively), Table 3. Fourteen (10.1%) patients had further surgeries for secondary wound closure (11; 78.6%) and exploratory laparotomy for residual intra- abdominal abscess (3; 21.4%). The duration of admission ranged from one to twenty-seven days with a mean (±SD) duration of 7.6 (±5.8) days (Table 3). Patients with complicated appendicitis stayed longer on admission compared to those who had uncomplicated appendicitis (χ^2^ = 58.8, p = 0.001). There was no mortality recorded from the disease or its treatment.

**Table 3:** **Post-operative complications, commencement of oral intake and duration of admission**

		**Type of appendicitis**		**Total N**	**p-value**
		**Uncomplicated N (%)**	**Complicated N (%)**		
*Post-operative complications	**Over-all rates**	1 (1.6)	34 (45.3)	35 (25.2)	
	Wound infection	1 (1.6)	29 (38.7)	30 (21.6)	
	Wound dehiscence	0 (0)	19 (25.3)	19 (13.7)	
	Intra-peritoneal abscess	0 (0)	3 (4.0)	3 (2.2)	
	Entero-cutaneous fistula	0 (0)	2 (2.7)	2 (1.4)	
	Incisional hernia	0 (0)	2 (2.7)	2 (1.4)	
	Adhesive bowel obstruction	0 (0)	3 (4.0)	3 (2.2)	
	Disseminated intravascular coagulopathy	0 (0)	1 (1.3)	1 (0.7)	
	Lobar pneumonia	0 (0)	1 (1.3)	1 (0.7)	
**Time to oral intake (days)**	0-2	59 (92.2)	34 (45.3)	93 (66.9)	*0.001*
	>2	5 (7.8)	41 (54.7)	46 (33.1)	
**Duration of admission (days)**	≤5	57 (89.1)	18 (24.0)	75 (54.0)	*0.001*
	>5	7 (10.9)	57 (76.0)	64 (46.0)	

*Some children had more than one post-operative complication.

## Discussion

In our study, appendicitis occurred more commonly in female children compared to their male counterpart (1.4: 1) contrary to most reported series and earlier study from this center [4,5,10]. However, Taiwo et al [11], and Uba and co-workers [12] had observed female preponderance in their series. All children in our study were 5 years and above. This corroborates global reports that the condition is rare in infants and pre- school children [2,5,13]. This may be explained by the paucity of lymphoid follicles in the wall of the appendix and the relative wideness of their appendiceal lumen [5]. Almost two thirds of the children in this series were seen in the second decade of life which is consistent with earlier reports on the disease [4,13,14].

Abdominal pain remains the most frequent early symptom, and was present in all the patients. It was followed by anorexia, vomiting and low grade fever which is also in tandem with earlier reports on the subject [4,12,14]. Vomiting in uncomplicated appendicitis is often once [15]. This observation we found to be true as most of those who vomited once or none at all had uncomplicated appendicitis. Of the 33 (23.7%) patients who presented with dehydration, 30 (90.9%) of them vomited more than once. This study showed that vomiting and dehydration were more associated with complicated appendicitis as compared with simple appendicitis. Children with acute appendicitis have poor oral intake, increased fluid losses due to fever and vomiting which make evaluation and management of their fluid and electrolyte status imperative in the peri-operative period. Intravenous re-hydration and analgesia should be provided. In our study, more than 80% of those with electrolyte abnormalities had complicated appendicitis. We were able to establish significant correlation between the presence of electrolyte abnormalities and complicated appendicitis. Thus serum electrolyte estimation should be done in patients who present with persistent vomiting and dehydration on admission [2,16]. The diagnosis of acute appendicitis was achieved mainly by clinical evaluation; ancillary investigations such as laparoscopy, ultrasound, and plain abdominal x-rays were deployed to aid in the diagnosis in some of our patients in whom clinical features were indeterminate. However in some instances, as earlier reported [4,5,17], we did not find these investigation results helpful. The Alvarado (MANTREL) scoring system is a useful tool which incorporates clinical features and basic haematologic indices to strongly suggest the likelihood that a right iliac fossa pain is due to acute appendicitis [18]. In children, this scoring system has been shown to correlate well with Computed Tomography scan findings and pathological review of appendix specimen [19]. The Alvarado score could not be computed in all patients due to incomplete records of the presence or otherwise of its components. This is a major limitation of this retrospective study. Also, the presence of leucocytosis and an elevated C-reactive protein have been shown to correlate with acute appendicitis and its severity [20-22]. Some researchers however opine that C - reactive protein does not improve the diagnostic accuracy in children [23]. In this study, C - reactive protein levels were not evaluated as the facility was not available within the study period and thus did not form part of our diagnostic workup.

It has been posited that acute appendicitis in children runs a rapid clinical course and that a delay in presentation of more than 12 hours from the onset of abdominal pain frequently results in perforation [4,12,14,24]. We found out that 84.0% of those with complicated appendicitis presented after 24 hours of onset of abdominal pain. There was a significant statistical relationship between perforation and delayed presentation (p < 0.05). In this series, the rate of perforation was high (71; 51.1%) compared to reports from other parts of Nigeria and sub-Saharan Africa. Uba et al [12] as well as Edino and co-workers [4] in Northern Nigeria reported perforation rates of 25.0% and 23.2% respectively while in South Africa, Chamisa [12] recorded about 34%. Our high perforation rate may be due to the delay in presentation of our patients. Like in previous studies, Lanz incision was our preferred approach for uncomplicated cases while midline and transverse incisions were reserved for those with suspected complicated appendicitis during clinical evaluation; [14,15,25]. We sometimes use the transverse supra-umbilical incisions in older children with pre-operative diagnosis of perforated appendix or generalized peritonitis to aid access to the sub -diaphragmatic spaces for the drainage of any probable collections. The location of the appendix and its length at surgery mirrored earlier findings with the preponderance of retro-caecal position, and length ranging from 4 to 25 cm [15,16]. The treatment protocol for acute appendicitis in our setting over the years has been open surgery after adequate resuscitation especially in those with moderate to severe dehydration. However, with the introduction of laparoscopic surgery into our clinical practice in the last four years, laparoscopic appendicectomy has been embraced and carried out in carefully selected cases with the hope that as we perfect the skill, laparoscopic appendicectomy will become a routine procedure in children with acute appendicitis.

Two patients had right hemi-colectomy with ileo-transverse anastomosis in our series due to perforation at the base of the appendix with edematous caecum. The fear here was that these patients could develop faecal fistula from failed repair of the perforation.

Most surgeons [4,5,14] advocate early diagnosis and prompt surgical intervention in children with acute appendicitis to avoid high post - operative complications and morbidity as was noted in this study. The overall post – operative complication rate was 25.2% and this was mainly due to delayed presentation. The high (21.6%) post-operative wound infection rate was comparable to findings in our sub-region [4,14] and was related to complications from delayed presentation. The skill of the surgeons can influence the nature and frequency of the post-operative complications reported in this series, however, the surgeons are experienced and the resident doctors that undertook the surgeries were not below the rank of senior registrars; who were closely supervised by specialists to prevent or mitigate such complications. We could not ascertain if hospital delay had an influence on the complications in this study due to its retrospective nature. There has been a steady decline in mortality from appendicitis to an acceptable rate of 0 to 2% [26]. Our current finding of zero mortality rate as against 2.3% reported earlier from our centre was in tandem with reports from Nigeria [4,5] and elsewhere [10,14].. Some advocates of early surgical therapy for acute appendicitis posit that this avoid high complication rates; and thus would accept a negative appendicectomy rate of about 10 to 25% [27]. The overall negative appendicectomy rate of 10.9% in this series is comparable to some reported studies from Nigeria [4] and other countries [14,17]. However, a much higher figure (52.3%) has been reported in the literature [12]. A larger number of misdiagnosis occurred predominantly in adolescent females in whom other pelvic conditions could mimic acute appendicitis. In such instances, ancillary investigations such as laparoscopy, abdominal ultrasound and computed tomography should be used as an adjunct to clinical examination to reduce the rate of negative appendicectomy [4,14]. However, some workers have argued that the rate of negative appendicectomy has not declined despite increasing use of such tests [28]. It is important to note that all children who had histologically normal appendices or reactive follicular hyperplasia appeared to have been cured of their ailment.

### Limitations

A major limitation of this study lies in its retrospective nature. Parameters for ascertaining Alvarado score were incomplete and long term follow up for late complications could not be ascertained. We believe that a prospective study validating Alvarado scoring system in predicting appendicitis in children in our setting would be necessary.

## Conclusion

Diagnosis of acute appendicitis in children in our setting is still based on high index of suspicion following clinical evaluation. This practice yielded an acceptable negative appendicectomy rate. More females than males had appendicitis in our study. Delayed presentation is associated with greater morbidity. No mortality occurred after appendicectomy in our series.
